# Erector spinae plane block for multimodal analgesia after wide midline laparotomy: A case report

**DOI:** 10.1097/MD.0000000000015654

**Published:** 2019-05-17

**Authors:** Seunguk Bang, Jihyun Chung, Woojin Kwon, Subin Yoo, Hyojung Soh, Sang Mook Lee

**Affiliations:** a Department of Anesthesiology and Pain Medicine, Daejeon St. Mary's Hospital, College of Medicine, The Catholic University of Korea, Daejeon; b Department of Anesthesiology and Pain Medicine, College of Medicine, The Catholic University of Korea, Seoul, Republic of Korea.

**Keywords:** analgesia, erector spinae plane block, laparotomy, nerve block, ultrasonography

## Abstract

**Rationale:**

The most commonly used regional techniques for analgesia following laparotomy thoracic epidural analgesia and paravertebral blocks are technically difficult to perform and carry a risk of severe complications. Recently, the erector spinae plane block (ESPB) has been reported to effectively treat neuropathic pain. The ultrasound-guided ESPB is an easily performed fascial plane block that can provide sensory blockade from T2–4 to T12–L1. Moreover, the ESPB reportedly blocks both the ventral rami of spinal nerves and the rami communicants, which contain sympathetic nerve fibres, through spread into the thoracic paravertebral space.

**Patient concerns:**

We report the case of a 35-year-old female patient who underwent excision of a larger ovarian mass via laparotomy with a wide, midline incision from the xiphoid process to the pubic tubercle.

**Diagnoses:**

They were diagnosed with mucinous cystadenoma originated from the right ovary and fallopian tube, and a right oophorectomy and salpingectomy were performed.

**Interventions:**

The ESPB was performed for postoperative pain control at the level of the T8 transverse process. Postoperative multimodal analgesia was provided according to the acute pain service protocol of our hospital. The patient was prescribed oral acetaminophen 175 mg every 6 hours and intravenous patient-controlled analgesia (PCA) with fentanyl 7 μg/mL. A 1:1 mixture of 0.75% ropivacaine (20 mL) and saline (20 mL) with epinephrine (1: 200,000) was manually injected through the indwelling catheter every 8 hours (20 mL per side).

**Outcomes:**

The first demand dose of fentanyl was administered at 9 hours and 39 minutes after the surgery. There were no reported resting pain scores >4, nor were any rescue analgesics needed during the first 5 postoperative days.

**Lessons:**

The ESPB provided highly effective analgesia as a part of multimodal analgesia after laparotomy with a wide midline incision.

## 1 Introduction

Thoracic epidural analgesia (TEA) and paravertebral blocks have been the most commonly used regional techniques for analgesia after laparotomy. However, these techniques are technically difficult to perform and have a relatively high risk of complications, including hypotension, bradycardia, motor blockade, urinary retention, epidural spread, and total spinal anesthesia. ^[1,2]^

Abdominal wall blocks, such as the transversus abdominis plane block and rectus sheath block, have been used as alternatives to TEA and paravertebral blocks; however, the efficacy of abdominal wall blocks for analgesia after laparotomy has not yet been established.^[3,4]^ Moreover, abdominal wall blocks do not block visceral pain, and they require multiple injections and a large volume of local anesthetic to block a wide range of dermatomes.^[3]^

Recently, the erector spinae plane block (ESPB) has been reported to provide effective analgesia after thoracic surgery, as well as analgesia for neuropathic pain.^[5,6]^ The ultrasound-guided ESPB is an easily performed fascial plane technique that can provide sensory blockade from T2–4 to T12–L2.^[5,7–11]^ Moreover, the ESPB anesthetizes both the ventral rami of spinal nerves and the rami communicants, which contain sympathetic nerve fibers, through spread into the thoracic paravertebral space.^[5,12]^ Here, we report a case in which continuous ESPB was used to provide highly effective analgesia after laparotomy with a wide midline incision.

## 2 Case report

Informed written consent for the publication of this report was obtained from the patient. And this report was approved by the Catholic University Hospital Institutional Review Board, Daejeon, Korea (DC18ZESI0040). A 35-year-old, 63-kg female patient was scheduled to undergo laparotomy for excision of a large ovarian mass. The patient had also experienced dyspnea due to pleural effusion resulting from the neoplasm. Preoperative pulmonary function tests revealed moderate restrictive lung disease with a forced expiratory volume in the first second (FEV1) of 1.17 L (45% of the predicted value) and a forced vital capacity (FVC) of 1.48 L (45% of the predicted value), which yielded an FEV1/FVC ratio of 79%. A preoperative computed tomography scan showed a large abdominal mass and a significant amount of pleural effusion with passive atelectasis in the right middle and lower lobes, as well as the left lower lobe.

**Figure 1:**
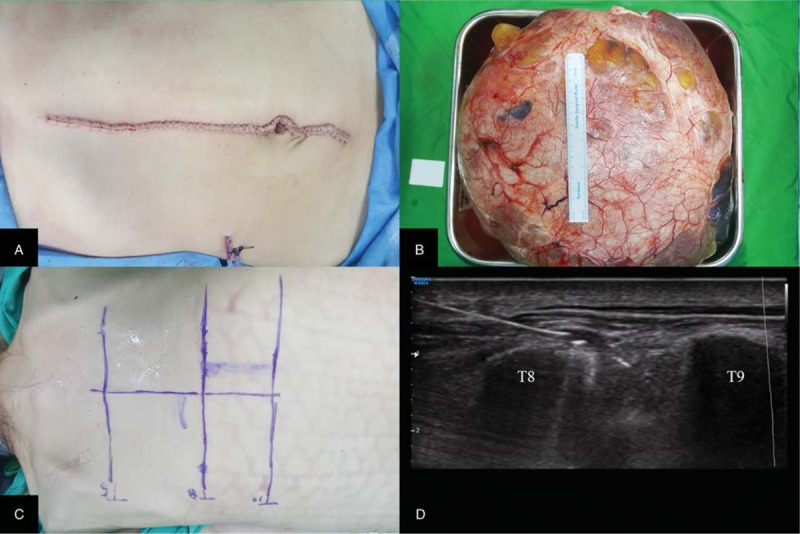
(A) An abdominal incision was made from the xiphoid process to the pubic tubercle. (B) The large ovarian mass was 36 cm × 34.5 cm × 17 cm (weight, 14 kg). (C) Prior to the block, landmarks were located and marked on the skin. (D) An ultrasound-guided erector spinae plane block was performed at the level of T8.

Following the induction of general anesthesia, a laparotomy was performed with a wide midline incision from the xiphoid process to the pubic tubercle (Fig. 1A). The mass originated from the right ovary and fallopian tube, and a right oophorectomy and salpingectomy were performed. The size of the mass was 36 cm × 34.5 cm × 17 cm and it weighed approximately 14 kg (Fig. 1B). During the procedure, 300 mL of ascites was also removed from the abdominal cavity.

After the surgical procedure, the patient was placed in the prone position, and an ultrasound-guided ESPB was performed at the level of T8 using an 18-gauge Tuohy needle (Fig. 1C). Using ultrasonography, we identified the T8 transverse process and 2 adjacent muscles (the trapezius and erector spinae). The Tuohy needle, which was connected to a syringe via an intravenous tubing extension, was then inserted in a cephalad-to-caudal direction toward the 2 muscles and the transverse process of T8. We confirmed that the needle was between transverse process and the erector spinae muscle by injecting 2 mL of saline under ultrasound visualization. We then injected a prepared mixture of 0.75% ropivacaine (10 mL) and saline (10 mL) with epinephrine 1:200,000 (Fig. 1D). A 19-gauge epidural catheter was then inserted through the Tuohy needle and advanced 2 cm beyond the needle tip under real-time ultrasound guidance. Catheter placement was confirmed by Doppler ultrasonography, whereas saline was injected through the catheter. The same procedure was followed to perform the ESPB on the contralateral side.

The total operative and anesthesia times were 2 hours and 10 minutes and 2 hours and 55 minutes, respectively. The patient's vital signs remained stable throughout the operation. At the end of the surgery, the patient was administered intravenous ketorolac 30 mg and fentanyl 50 μg. Postoperative multimodal analgesia was provided according to the acute pain service protocol of our hospital. The patient was prescribed oral acetaminophen 175 mg every 6 hours and intravenous patient-controlled analgesia (PCA) with fentanyl 7 μg/mL. The PCA settings were as follows: a background infusion of 1 mL/h and a bolus dose of 3 mL with a lockout time of 7 minutes. A 1:1 mixture of 0.75% ropivacaine (20 mL) and saline (20 mL) with epinephrine (1: 200,000) was manually injected through the indwelling catheter every 8 hours (20 mL per side). The patient's resting and dynamic (coughing, deep breathing) pain scores after surgery were assessed using the visual analogue scale (VAS). If the VAS score was >4, then the protocol called for the patient to receive 25 mg of intravenous tramadol and 25 mg of intravenous meperidine as rescue analgesics.

In the postanesthesia care unit, the patient's resting and dynamic pain scores were 1 to 2 and 3 to 4, respectively. Two hours postoperatively, the patient showed decreased sensory by pinprick test from T3 to T11 on the left and from T4 to T10 on the right at the midclavicular line. The intravenous PCA was discontinued on postoperative day 3 per the acute pain service protocol. The patient's fentanyl consumption and demand were tracked using the PCA pump (Table 1). The patient received her first demand dose of 21 μg of fentanyl through the PCA at 9 hours and 39 minutes after the surgery. There were no reported resting or dynamic pain scores >4, nor were any rescue analgesics needed during the first 5 postoperative days. At her 1-month follow-up visit, the patient had no residual sensory or motor deficit, nor did she complain of symptoms suggestive of a neurological injury.

**Table 1:**
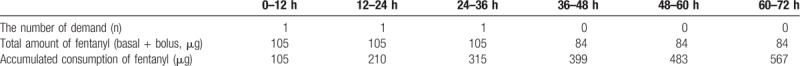
The amount of fentanyl consumption.

## 3 Discussion

Pain after laparotomy consists of both visceral pain and somatic pain from the abdominal wall. The visceral pain is conducted to the spinal cord through the sympathetic chain, whereas somatic pain is conducted through the T7–L1 spinal nerves.^[3]^ TEA and paravertebral blocks are advantageous in that they can provide both visceral and somatic analgesia; however, these techniques are technically difficult to perform and have a relatively high risk of complications.

Alternatives to TEA and paravertebral blocks include abdominal wall blocks, which block the thoracoabdominal spinal nerves. However, abdominal wall blocks, such as the transversus abdominis or rectus sheath block, do not provide a wide range of sensory blockade. Moreover, these blocks require multiple injections and usually need to be performed in combination with another type of abdominal wall block. In addition, it is often difficult to perform a rescue abdominal wall block due to surgical tissue disruption and the presence of subcutaneous air and/or a wound dressing after surgery.

The ESPB is a relatively simple technique that uses easily identifiable sonographic landmarks.^[8]^ Moreover, a catheter can easily be inserted into the plane following once the tissues have been separated by a saline injection. Unlike abdominal wall blocks, the ESPB can provide a wide range of sensory blockade of both the upper and lower abdomen. In addition, it is reported that the ESPB can provide both somatic and visceral analgesia, and it has a lower risk of complications than TEA or paravertebral blocks.^[5,7,12]^ In addition, the ESPB can be performed regardless of surgical tissue disruption and the presence of subcutaneous air or a wound dressing.^[13]^

The patient in this case received her first PCA demand dose about 10 hours after the surgery, and the numbers of bolus demand were 2 and 1 during 24 and 48 hours. Chung et al^[14]^ reported that the amount of fentanyl consumption at 24 hours was 286 μg after single port laparoscopic adnexectomy. Her fentanyl consumption of 210 μg at 24 hours is quite low, considering the laparotomy was performed with wide midline incision. It was indicating that the ESPB provided effective postoperative analgesia in this case. Other report showed that ESPB can be a good option for multimodal analgesia after abdominal surgery by reducing opioid consumption and controlling the postoperative pain.^[13]^

We think the mechanism is twofold: spread through the bony gap, and penetration through porous tissue around the superior costotransverse ligament. Some cadaveric studies have shown the range of the ESPB to spread to the ventral rami of multiple levels, the neural foramina, and the epidural spaces, although another study even reported that the range of the ESPB was mostly confined to the dorsal ramus and only about 10% was spread to the ventral ramus or the dorsal root ganglia. ^[5,15–17]^

In conclusion, the ESPB is technically easy to perform and utilizes easily identifiable sonographic landmarks, which likely decreases the risk of complications. Therefore, we believe that the ESPB can be a safe and effective part of multimodal analgesia after laparotomy. Further case reports and prospective, randomized studies are needed to verify the efficacy of the ESPB for analgesia after laparotomy.

## Author contributions

**Conceptualization:** Seunguk Bang.

**Data curation:** Seunguk Bang, Jihyun Chung, Woojin Kwon, Subin Yoo, Hyojung Soh.

**Visualization:** Woojin Kwon.

**Writing – original draft:** Seunguk Bang, Jihyun Chung, Subin Yoo, Hyojung Soh.

**Writing – review and editing:** Seunguk Bang, Sang Mook Lee.
